# Effect of Plant-Based Diets on Gut Microbiota: A Systematic Review of Interventional Studies

**DOI:** 10.3390/nu15061510

**Published:** 2023-03-21

**Authors:** Shaneerra Raajlynn Kaur Sidhu, Chin Wei Kok, Thubasni Kunasegaran, Amutha Ramadas

**Affiliations:** Jeffrey Cheah School of Medicine and Health Sciences, Monash University Malaysia, Jalan Lagoon Selatan, Bandar Sunway 47500, Selangor Darul Ehsan, Malaysia

**Keywords:** plant-based diet, gut microbiome, metabolome

## Abstract

Plant-based diets have grown increasingly popular across the globe, mainly for their health and environmental benefits. Several studies have identified a link between plant-based diets and the decreased risk of developing cardiovascular diseases, obesity, and other health issues. We systematically reviewed human interventions to identify the relationship between various plant-based food items and the gut microbiome, alongside the biochemical and anthropometric measurements as secondary findings. The study selection process was completed using the COVIDENCE platform. Overall, 203 studies were identified, of which 101 were chosen for title and abstract screening by two independent authors. Following this process, 78 studies were excluded, and the full texts and the reference lists of the remaining 23 records were reviewed using the review eligibility criteria. A manual search yielded five additional articles. In the end, 12 studies were included in the systematic review. We found evidence for short- to moderate-term beneficial effects of plant-based diets versus conventional diets (duration ≤ 13 months) on gut microbiome composition and biochemical and anthropometric measurements in healthy participants as well as obese, cardiovascular, and rheumatoid arthritis patients. However, contradictory results were observed for Enterobacteriaceae, at the family level, and for Faecalibacterium and Coprococcus, at the genus level, of gut microbiome composition. The relationship between plant-based diets and the gut microbiome, alongside their underlying metabolic and inflammatory effects, remains largely unexplored. Hence more interventional studies are needed to address these questions.

## 1. Introduction

Plant-based or vegetarian diets incorporate most or all of the food derived from plant-sourced origins while excluding different combinations of products of animal origin, including red meat, fish, poultry, eggs, and dairy [1,2]. Additionally, the flexitarian or semi-vegetarian diet includes a small percentage of meat, dairy, poultry, or seafood into a mainly plant-rich diet, allowing for a more significant percentage of plant products to be incorporated into an individual’s diet without the need to remove existing varieties of food [3]. Pescatarians, however, avoid meat and poultry altogether from their diet but consume fish and seafood. However, lacto-ovo vegetarian diets include eggs and dairy but exclude meat, fish, poultry, and seafood. On the other hand, vegans strictly do not consume meat or purchase by-products of animals that involve animal testing or the inhuman torture of animals.

Plant-based diets are gaining popularity worldwide, mainly due to their health benefits, environmental concerns, and religious following. The most prevalent plant-based diet practiced is vegetarianism, with an estimated 1.5 billion followers worldwide [4]. With Asia being the leading continent for plant-based diet adoption, it is estimated that almost one-fifth of the Asian population embraces vegetarianism [5]. More predominantly, India has the highest vegetarianism following at nearly 40% of the population [5]. Moreover, vegetarianism has been linked to religious followings that promote nonviolence and respect for all living beings, including Hinduism, Sikhism, Jainism, and Buddhism [3]. Religious motivations for plant-based lifestyles are due to beliefs mainly based on the ideology that animal slaughter consumption is disrespectful, unethical, or immoral.

Subsequently, the increase in the number of plant-based diet consumers is accredited to the increasing social awareness of animal rights and animal cruelty when producing food sourced from animals [3]. Furthermore, factors, such as weight maintenance, mental well-being, food allergies, and family influences, can also be factors in incorporating plant-based diets [3].

At the height of the COVID-19 pandemic, many American households purchased plant-based foods, including milk and meat alternatives. This widespread adoption may be attributed to the heightened awareness of the environmental costs of producing food spurred by the pandemic. Worldwide acceptance of plant-based diets can drastically decrease the carbon emissions produced, mainly through rearing animals and livestock. Increasing awareness of the social and environmental effects of greenhouse gases, such as methane and carbon dioxide, on climate change has encouraged consumers to be more plant-based food-reliant in hopes of playing their part in reducing their carbon footprint [3].

Plant-based protein sources are also increasing in developed countries via foods such as tempeh, lentils, and quinoa. Many believe that plant-based foods are healthier and beneficial to our gut microbiome, as they generally undergo less chemical processing and are more natural. In general, plant-based diets are lower in saturated fats and high in fiber and phytochemicals, contributing to lower concentrations of blood low-density lipoprotein cholesterol [6]. Thus, integrating these diets has decreased the risk of followers developing cardiovascular diseases, obesity, diabetes, hypertension, and certain cancers [6]. However, more research is needed surrounding the effectiveness and readiness of plant-based protein absorption and how the gut microbiota influences this.

The human gut microbiota, or natural microbial community, refers to the ecosystem of organisms in the digestive tract of humans and animals. It comprises primarily bacteria, archaea, and microscopic eukaryotic organisms but includes viruses, fungi, and protozoa. An estimated three trillion microbes comprise each individual’s gut microbiota profile [7]. It is believed that the microorganisms making up our gut microbiota play an essential role in our health, predicting the risks of developing certain gastrointestinal diseases. Environmental factors, such as diet, and the combination of our dietary habits directly affect the composition of our gut microbiota due to their influence on the bacteria ecosystem [8]. Literature suggests that adopting plant-based diets increases beneficial bacteria in our gut, such as the phylum Bacteroidetes, leading to gut and overall health promotion [9].

However, a review has shown that certain diet manipulation may result in gut dysbiosis, possibly negatively influencing disease progression via altering the release of metabolically active products by the gut microbiome [10]. Therefore, we aimed to conduct a systematic review of interventions that report the effects of various plant-based diets on gut microbiota

## 2. Materials and Methods

The 2020 Preferred Reporting Items for Systematic Reviews and Meta-Analyses (PRISMA) statement [11] and checklist was used to perform a systematic review (Supplementary Table S1) [11]. The protocol for this systematic review has been registered with PROSPERO (CRD42022345680) and can be accessed via www.crd.york.ac.uk/prospero/ (accessed on 27 July 2022).

### 2.1. Data Sources and Search Strategy

We considered intervention studies, including randomized controlled trials, non-randomized trials, and pre–post interventions that reported on the effects of plant-based diets on gut microbiota. The study population included adults on plant-based diets either at the time of recruitment or who were counseled to eat a plant-based diet within a specific timeframe. The gut microbiota must be quantified from stool samples using any sequencing approach targeting the 16s ribosomal RNA gene. Studies that use liquid gas chromatography to detect microbial metabolites’ roles were also included. Studies that reported only a single type of food were excluded.

A literature search of peer-reviewed publications was performed using the following electronic bibliographic data sources: Ovid Medline, Scopus, PubMed, and Embase. The search strategy was built using the following MeSH terms and keywords: (plant-based) OR (vegetarian) OR (vegan) AND (gut microbe) OR (probiotic) OR (prebiotic) AND (intervention). The search was limited to “article” where possible. We imposed no language restrictions. We also conducted manual research for relevant papers, using the reference lists of the included studies and previous reviews.

The search strategy conducted for all databases is shown in Supplementary Table S2.

### 2.2. Study Selection

We excluded duplicate studies and conducted the study selection process using the COVIDENCE platform. Two authors (S.S. and C.K.W.) independently screened the retrieved references’ titles and abstracts and evaluated the full texts according to the review’s eligibility criteria. A third author (A.R.) resolved any conflicts during both screening stages. Studies were included if they met the following criteria: (1) intervention studies on the effects of plant-based diets on gut microbiota among adults; (2) metagenomics sequencing analysis was carried out. Non-peer-reviewed publications, such as book chapters and conference proceedings, were excluded. Studies reporting on adults consuming a single type of food were also excluded.

### 2.3. Data Extraction and Synthesis

The authors independently extracted relevant information from the included studies using Google Sheets: author, country, study design, number of participants, and primary and/or secondary outcomes. The primary outcomes were gut microbiota changes, including relative bacterial abundance and diversity (α and β) levels. Other outcomes were changes in metabolic parameters and weight. The characteristics of the studies were summarized. Data on the types of gut microbiota and their correlation with outcomes in adults on plant-based diets, if any, were qualitatively synthesized.

### 2.4. Quality Assessment and Risk of Bias

The National Institutes of Health (NIH) quality assessment tool for randomized control and pre–post studies was used to assess the included studies’ methodological quality [12]. The Risk of Bias in Non-randomized Studies of Interventions (ROBINS-I) tool was used for non-randomized studies [13]. The quality was assessed based on the study population, eligibility criteria, sample size justification, timeframe to investigate the effect, exposure, and outcome details, and other sources of bias. Each included study was classified as being of good, fair, or poor quality. Two reviewers (S.R.K.S. and C.W.K.) independently assessed all analyses for methodological quality.

## 3. Results

### 3.1. Study Selection

**Figure 1:**
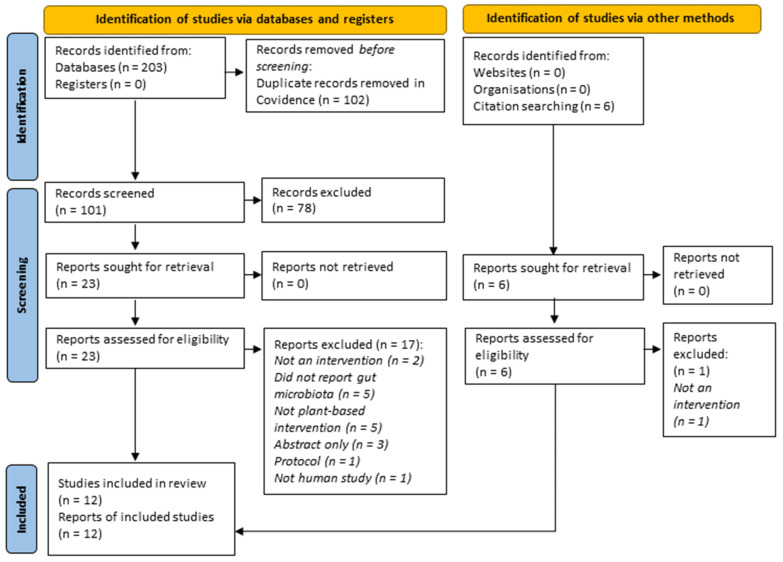
PRISMA 2020 flow chart detailing the study selection process.

The database search found 203 records. In total, 101 titles and abstracts were evaluated after 102 duplicate entries of articles were removed. Subsequently, 23 full texts were reviewed using the review eligibility criteria. A manual search yielded six additional articles; one was excluded after not fulfilling the criteria. Most reports were excluded because they did not report on a plant-based intervention (n = 5), did not report findings on gut microbiota (n = 5), or were conference abstracts (n = 3). Finally, 12 studies matched the inclusion criteria and were included in the systematic review (Figure 1).

### 3.2. Study Characteristics

The summary of the main characteristics of the included studies is displayed in Table 1. The comprehensive details of the studies are included in Supplementary Table S3. All 12 included studies were assigned to interventional study design. Two studies were published in the 1990s [14,15], three studies were published between 2010 and 2018 [16,17,18], and all other studies were retrieved from the year 2020 to 2021 [19,20,21,22,23,24,25]. The geographical origins of the included studies were diverse. Ten studies were conducted in Europe, particularly the United Kingdom [24], Sweden [18,21], Finland [14,15], Germany [23], and Italy [19]; four were from the United States of America [19], and one study was conducted in Asia (Korea) [17].

The sample sizes ranged from 6 [17] to 168 [20], with a total of 583 participants comprised of healthy, obese, cardiovascular risk, and rheumatoid arthritis individuals. All studies reported the inclusion of men and women, except one [18] that did not report on sex. The ages of the participants ranged between 21 and 61 years. The duration of the intervention varied from 5 days to 13 months.

Six studies reported vegan dietary intervention [14,15,17,20,23,25]. Three studies investigated the effect of ovo-lacto vegetarian dietary interventions [18,19,21] and plant-based diets [16,22,24] on gut microbiota composition. Six studies [18,20,22,23,24,25] performed 16s-RNA sequencing to detect the gut microbiota composition, except for two studies that used gas–liquid chromatography to investigate the role of microbial metabolites [14,15]. Four studies [16,17,19,21] used both methods.

### 3.3. Study Quality Assessment

The mean score on the NIH Quality Assessment Scale for the intervention studies was 58.2% (21.4–92.8%). Four studies had good quality [14,15,23,24], four had acceptable quality [19,20,21,25], and one had poor quality [18] (Supplementary Table S4). The mean score on the NIH Quality Assessment Scale for the pre–post studies [17,22] was 50.0%, and both had fair quality (Supplementary Table S5). Assessed using the ROBINS-I tool, the work completed by David et al. [16] was found to have an overall low risk of bias (Supplementary Table S6).

### 3.4. Gut Microbiota Composition in Vegetarian/Vegan Diets

**Figure 2:**
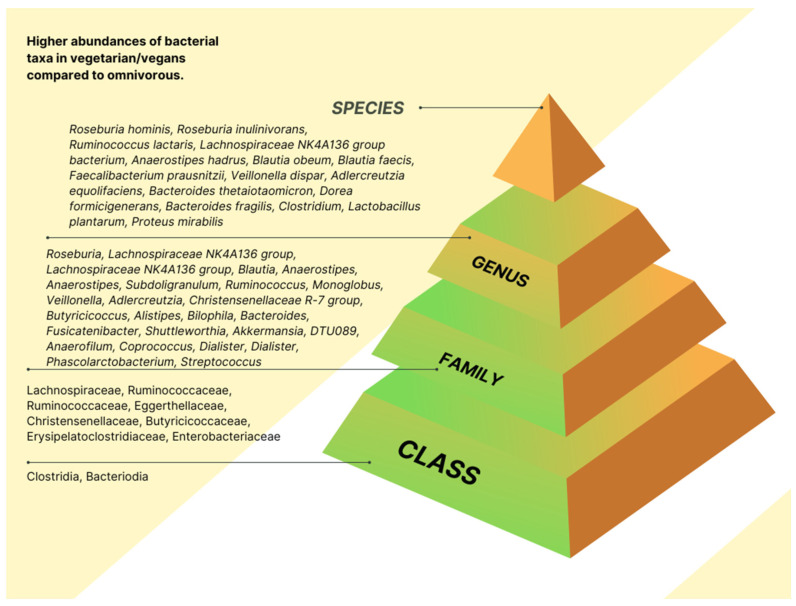
Higher abundances of bacterial taxa in vegetarian/vegan intervention diets. The figure was created and edited from the images available on Canva.com using a Pro Content License.

**Figure 3:**
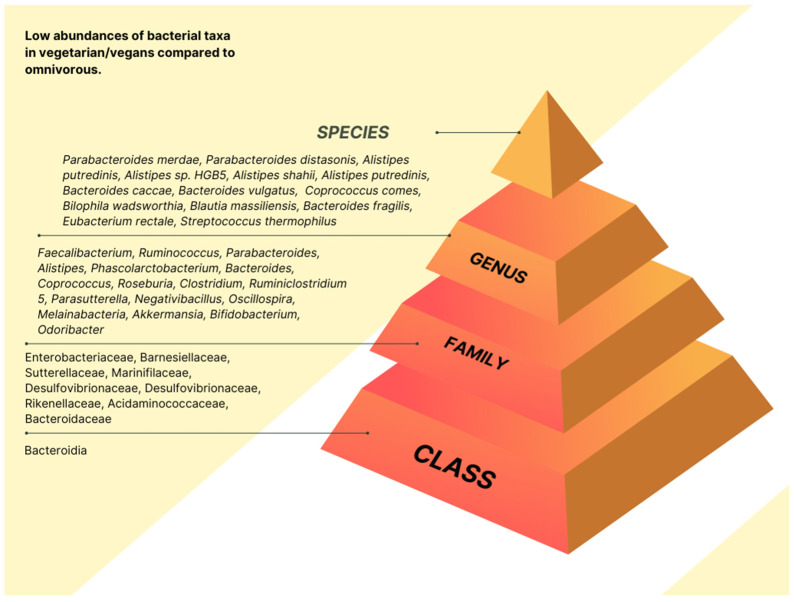
Low abundance of bacterial taxa in vegetarian/vegan intervention diets. The figure was created and edited from the images available on Canva.com using a Pro Content License.

The link between diet and microbiota composition in vegan or vegetarian intervention is displayed at the class, family, genus, or species level, according to the information retrieved from the included studies. The statistically increased abundances of taxa are presented in Figure 2 and Supplementary Table S7. The statistically reduced taxa abundances are shown in Figure 3 and Supplementary Table S8.

At the family level, contradictory results were observed for Enterobacteriaceae. One study [22] found higher abundances of Enterobacteriaceae in the adult with CVD risk with a plant-based diet and low abundances in vegans [17]. Four studies found statistically significantly increased levels of Ruminococcaceae in vegan [20,23] and plant-based diets [22,24]. Bacteroidaceae was reduced significantly in vegan [23] and plant-based diets [22]. Ahrens et al. [22] reported a significant increase in Lachnospiraceae, Ruminococcaceae, Monoglobaceae, Eggerthellaceae, Christensenellaceae, Butyricicoccaceae, and Erysipelatoclostridiaceae in individuals who followed a six-day plant-based diet. The authors [22] also observed a significant reduction in Barnesiellaceae, Sutterellaceae, Marinifilaceae, Marinifilaceae, Tannerellaceae, Rikenellaceae, and Acidaminococcaceae.

The same applies to the genus *Faecalibacterium*, which tended to be less abundant in vegans [23] and had a high abundance in plant-based diets [22]. Genus *Alitispes* was increased in animal-based [16] and vegan diets [23]. In contrast, Ahren et al. [22] reported a reduced abundance of *Alistipes* in individuals with a plant-based diet. Bacteroides were found to be statistically increased in animal-based [16] and vegan diets [17], compared to a significant reduction in plant-based diets [22]. The genus *Faecalibacterium* and *Coprococcus* were higher in vegans [23] and significantly reduced in plant-based diets [22]. The plant-based diet [22], vegan diet [23,25], and ovo-lacto vegetarian diet [18] had increased abundances of the genus *Ruminococcus.* Finally, the genus *Roseburia* was found at statistically increased levels in the plant-based diet [22] and ovo-lacto vegetarian diet [18]; however, the genus abundance was decreased in the animal-based diet [16] and vegans [23]. The other genus was shown to be increased and decreased significantly based on one individual study (Supplementary Tables S7 and S8).

The *Faecalibacterium prausnitzii* sp, was significantly increased in plant-based [22,24] and vegan diets [20,23]. The *Bacteroides fragilis* was markedly increased in vegans [17,23], but, Kahleova et al. [20,25] also reported a significant reduction among individuals with a low-fat vegan diet. The other species were shown to be increased and decreased significantly based on one individual study (Supplementary Tables S7 and S8).

### 3.5. Association of Plant-Based Diets and Gut Microbiota on Metabolic, Inflammatory, and Body Composition in Healthy and Unhealthy Subjects

**Figure 4:**
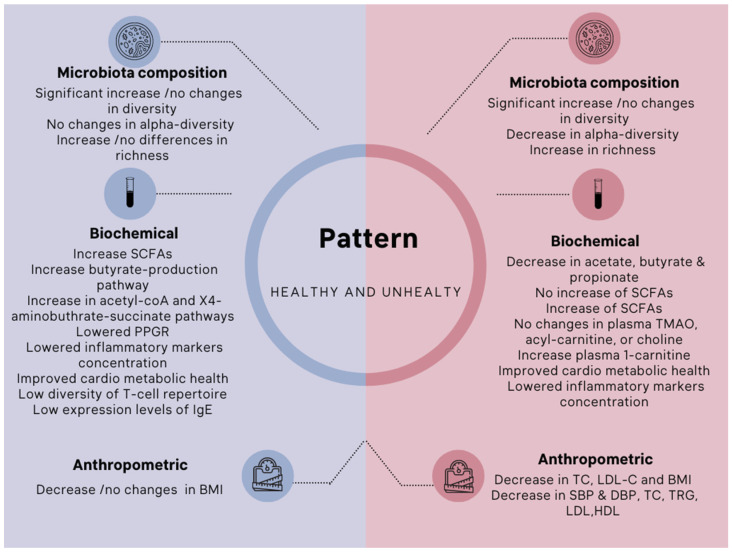
The influence of vegan/vegetarian diets on gut microbiota composition and biochemical and anthropometric levels in the healthy and unhealthy (metabolic and autoimmune disturbed) subjects. Note: short-chain fatty acids (SCFAs), postprandial (post-meal) glycemic response (PPGR), systolic blood pressure (SBP), diastolic blood pressure (DBP), total cholesterol (TC), triglycerides (TRG), low-density lipoprotein (LDL), high-density lipoprotein (HDL), low-density lipoprotein-cholesterol (LDL-C), body mass index (BMI). References under healthy pattern for microbiota composition-Significant increase [18]/no changes in diversity [16,23,24], no changes in alpha-diversity [16], increase [16]/no differences in richness [16,23,24]; for biochemical component- increase in SCFAs [23], increase in butyrate-production pathway and acetyl-coA and X4-aminobuthrate-succinate pathways [24], lowered PPGR [24], lowered concentration of inflammatory markers [18,22,23], improved cardiometabolic health [23], low diversity of T-cell repertoire and low expression levels of IgE [18]; for anthropometric-decrease in BMI [21], mo changes in BMI [18,24]. References under unhealthy pattern for microbiota composition-significant increase in diversity [19,21,22,25], no changes in diversity [14,20], decrease in alpha-diversity [21], increase in richness [14,21,22]; for biochemical component–decrease in acetate, butyrate & propionate [14], no increase of SCFAs [19], increase of SCFAs [21,22], no changes in plasma TMAO, acyl-carnitine, or choline [21], increase plasma 1-carnitine [21], improved cardiometabolic health [22], lowered inflammatory markers concentration [22]; for anthropometric-decrease in TC, LDL-C and BMI [17,20,21,22,25], decrease in SBP & DBP, TC, TRG, LDL, HDL [21,22]. The figure was created and edited from the images available on Canva.com using a Pro Content License.

Various microbial metabolites have favorable health benefits. These include anti-inflammatory, immunomodulatory, systemic anti-obesogenic, antihypertensive, hypocholesterolemic, antiproliferative, and antioxidant effects [26]. These postbiotic effects depend on the composition and substrates of the microbiota and are mainly influenced by diet. They arise from the control of gene expression, metabolism, and intestinal function. The twelve studies included in this review reported various health benefits from a range of participants (Figure 4).

### 3.6. Healthy Adults

Four studies reported the effects of microbial metabolites in healthy subjects [16,18,23,24]. It was shown by David et al. [16] that the gut microbiota rapidly adapted in response to a plant-based or animal-based diet. The changes were visible within five days of the new diet. There was a bigger shift in the composition of the gut microbiota from species that metabolize dietary plant polysaccharides to those that metabolize bile acids, suggesting that the animal-based diet has a more significant effect on modifying the gut microbiota than the plant-based diet. Remarkably, the one vegetarian in the study who switched to an animal-based diet had the most significant decrease in *Prevotella.*

According to Zhang et al., long-term vegetarianism was related to a less diverse T-cell repertoire [18]. Long-term vegetarians also exhibited reduced levels of IgE, a crucial allergy-related immunological indicator. The intervention and control groups did not differ in body mass index (BMI). Similarly, Toribio et al. [24] found no significant differences between the omnivore diet and a vegetarian or vegan one concerning age, gender, BMI, or weight.

Kohnert et al. [23] established a link across all clinical markers of the genus *Odoribacter* in the vegan group and the genus *Clostridium* in the Mediterranean diet group. All of the branched-chain amino acids (VAL, ILE, and LEU) were shown to have a positive correlation with *Megamonas* in vegans. In contrast, *Coprococcus* and *Dorea* were found to have a negative correlation. Within the Mediterranean diet group, an inverse association existed between branched-chain amino acids and *Dorea.* Further, a cluster analysis using phylogenetic information revealed an intriguing divergence between two sample groups, which were designated as Phylo1 and Phylo2. The differentiation of enterotypes did not explain the splits sufficiently, nor did any of the evaluated sociodemographic or clinical factors.

### 3.7. Metabolic Diseases

The role of a plant-based diet in influencing obesity and inflammation was explored by Kim et al. [17], Pagliai et al. [19], and Kahleova et al. [20,25]. Kim et al. [17] demonstrated the positive effects of a vegan diet on the link between gut microbiota and metabolic syndrome. The authors [17] included six obese, diabetic, and/or hypertensive individuals. The results showed lower blood glucose levels, body weight, triglycerides, total cholesterol, LDL-cholesterol, and hemoglobin A1c levels, following a month of vegan diet intervention. The number of *Firmicutes* was drastically reduced, and the abundance of *Bacteroidetes* was dramatically increased, due to the vegan diet therapy-induced changed gut microbiota. Even though the ratio of Firmicutes to Bacteroidetes changed, the host’s enterotype did not change. This is because *Prevotella* and *Bacteroides*, which break down plant polysaccharides, grew in response to the vegan diet. In particular, the Enterobacteriaceae family of bacteria—known to cause chronic, low-grade inflammation—was found to be reduced in those following a vegan diet.

Pagliai et al. [19] showed no statistically significant differences in short-chain fatty acid (SCFA) production between the Mediterranean and vegetarian diets. In contrast, a vegetarian diet produced less propionic acid and more isobutyric and isovaleric acids. Correlation analyses revealed a possible association between changes in taxa and variations in clinical and biochemical indicators, including anthropometric parameters, metabolic variables, and inflammatory variables, which were altered by the two diets.

The relative abundance of Proteobacteria decreased after a 16-week vegan diet, while the ratio of Bacteroidetes to Firmicutes remained unchanged [20]. The Firmicutes to Bacteroidetes ratio increased (*p* = 0.04), and the butyrate-producing bacteria decreased (*p* = 0.02) on the Mediterranean diet [25]. Kahleova et al. [20,25] observed a drop in body weight in the vegan diet intervention group, mainly attributed to a decrease in fat mass and visceral fat. The indicator of insulin sensitivity rose among vegans. They reported an increase in *Faecalibacterium prausnitzii*, adversely linked with weight, fat mass, and visceral fat alterations. *Bacteroides fragilis’* relative abundance dropped in both groups (omnivorous and vegan), but less in the vegan group. This species was also found to be inversely connected with weight, fat mass, and visceral fat alterations, and positively correlated with insulin sensitivity.

Djekic et al. [21] have established a positive link between 1-carnitine metabolism and cardiovascular disease (CVD) risk following a vegan diet intervention. Patients with ischemic heart disease receiving optimal medical care benefitted from switching from a ready-made meat diet to an isocaloric ready-made vegetarian diet under an individually designed diet plan. Subjects who consumed a vegetarian diet for the study had considerably lower levels of oxidized low-density lipoprotein cholesterol, low-density lipoprotein cholesterol, total cholesterol, and body mass index than those who consumed meat as their primary source of protein. Subjects on the vegetarian diet had a lower relative abundance of fecal microbial taxa and plasma compounds linked with metabolic disease, particularly cardiovascular disease, than those on a meat diet.

A separate trial evaluated the effects of a combined plant-based diet and exercise intervention on CVD risk variables, including blood pressure and lipid profiles. Ahrens et al. [22] reported a statistically significant decrease in blood pressure and lipid profile. Without weight loss, reductions in blood pressure, total cholesterol, and triglycerides were also observed. Significant increases in butyrate producers, notably Lachnospiraceae and *Oscillococcales*, were identified. Substantial changes in relative abundance were seen within individuals, such as an increase in Lachnospiraceae, Ruminococcaceae, *Faecalibacterium prausnitzii,* and diversification with richness. Changes in the microbiota were substantially linked to variations in the BMI, blood pressure, cholesterol, high-sensitivity C-reactive protein, glucose, and trimethylamine N-oxide (TMAO).

### 3.8. Inflammatory Diseases

Studies of adults with rheumatoid arthritis showed that a one-month switch to a vegan diet was enough to significantly change the fecal microbiota, as measured by the stool sample gas–liquid chromatography profiles of bacterial cellular fatty acids [14,15]. This suggests that a vegan diet may cause a rapid change in the gut profile. Peltonen et al. [14] conducted research on 53 rheumatoid arthritis patients. They discovered a significant difference in intestinal microbiota following a one-year transition from a conventional diet to a vegan and then lactovegetarian diet.

To find out more about the role of fecal microbiota in affecting rheumatoid arthritis, 43 rheumatoid arthritis patients were randomly given either a raw vegan diet high in lactobacilli or a diet with meat, fish, and vegetables [15]. After a month on a vegan diet, the fecal microbiota of the 18 study participants who adhered to it significantly differed from that of the omnivore control group. Notably, the vegan diet also caused some rheumatoid arthritis patients to have less disease activity. This led the authors to conclude that changes in the fecal microbiota and its disease activity are caused by diet [15].

## 4. Discussion

This systematic review is the first to assess the association between vegan and vegetarian diets on gut microbiota composition and human health outcomes using the retrieved data from twelve intervention studies. Several review articles published during the recent decade highlighted distinct microbiome compositions in vegans and/or vegetarians [9,27,28,29]. Those articles focused on observational studies and lacked intervention studies to prove the associations between gut microbiota composition and vegan or vegetarian diet on human health benefits. The extracted results on the differences in microbiota composition between the type of diets suggest the presence of more bacteria that break down the fiber in vegan/vegetarian compared to fat-protein metabolizing bacteria in the animal-based diet.

The vegan/vegetarian diets are rich in dietary fiber fermentation products and other carbohydrates that produce SCFAs. The fecal levels of these metabolites are strongly associated with fruit, vegetable, and legume intake. Thus, their levels increase dramatically in individuals who adopt a plant-based diet. Intriguingly, a diet high in fruit, legumes, and vegetables, i.e., the plant-based diet, was associated with a rise in SCFAs in our included studies [21,22]. The included study also showed a negative correlation with pro-inflammatory cytokines [19].

On the other hand, an animal-based diet resulted in significantly lower levels of carbohydrate fermentation products and a higher concentration of amino acid fermentation products [16]. In contrast, Kim et al. [17] observed a low fecal SCFA concentration in their strict vegetarian diet subjects, mainly due to the weight loss observed in the subjects and the high content of low fermentable non-starch polysaccharides in their diet [30]. Interestingly, the plant-based diet [24] also observed an increase in SCFA-butyrate metabolizing pathways; however, a study needs to be conducted on a larger scale to confirm the finding.

Butyrate has multiple physiological activities, including providing energy to colonocytes and improving the intestinal barrier via the upregulation of tight junctions. Butyrate lowers systemic inflammation by preventing lipopolysaccharide (LPS) from crossing the intestinal wall and entering the bloodstream [31]. The intervention studies included in the review have reported an increase in the butyrate-producing bacteria, such as Ruminococcaceae, Lachnospiraceae, *Coprococcus*, *Roseburia*, *Blautia*, *Alistipes,* and *Faecalibacterium prausnitzii*. Substantial evidence shows that acetate, propionate, and butyrate have a preventive effect against various diseases, including type 2 diabetes, inflammatory bowel disease, and immunological diseases. SCFAs, for instance, have been demonstrated to boost immunity in the host [32]. They are also crucial for neuron function and maturation and for maintaining the blood–brain barrier function [33]. SCFAs also promote metabolism and play a significant role in preventing or treating obesity [34,35]. The considerable body weight reduction observed in vegan and vegetarian intervention diets strongly supports the claim.

Another possible beneficial linkage observed in these intervention studies was the correlation of TMAO measurement with the relative abundance of specific microbes. Djekic et al. [21] observed a significant correlation between the genus Bifidobacterium and several species of Lachnospiraceae and Ruminococcaceae. They also observed a reduction in the plasma L-carnitine (a metabolite found in red meat) among subjects with ischemic heart disease who adhered to the vegan diet intervention. Red meat consumption has been associated with elevated TMAO levels, which increases the risk of cardiovascular disease and inflammatory bowel disease [36,37]. The study [21] suggests cardiometabolic benefits (reduced oxidized LDL-C, TC, and body weight) in vegetarian diet interventions, supporting the beneficial role of gut microbiota in modulating the biochemical parameters associated with CVD risk. However, Ahrens et al. [22] did not detect significant differences in plasma TMAO concentration within the intervention period with a plant-based diet in individuals with moderate-to-high CVD risk. We postulated that the team’s six-day intervention [22] might be insufficient to observe the changes in relation to the TMAO concentration compared to the 4-week intervention period utilized by Djekic et al. [21].

The presence of SCFAs and TMAO metabolites in the plasma of vegan and vegetarian diet intervention suggests gut microbiota’s role as a modulator of the diet–host interaction. Despite the identified mechanism involved, the interaction of gut microbiota with other metabolites, such as plant-derived polyphenols, vitamins, isothiocyanates, and intestinal aryl-hydrocarbon receptor ligands, is lacking. Thus, we could not provide a comprehensive conclusion about the mechanism behind gut microbiota interactions in vegan or vegetarian diets. Therefore, more interventional studies are warranted to evaluate the possible linkage between gut microbiota composition and vegan/vegetarian diet interventions in terms of the overall health benefits.

## 5. Limitation

There were some limitations observed in the included studies. Most studies had a low number of participants, which might have insufficient power to the study result. Besides that, only one study [17] included participants from the Asian continent, thus calling into question the results’ generalizability to the Asian population owing to different dietary intake, environmental exposure, or lifestyle habits. Antibiotics [38,39,40] and other drugs [41,42] are known to change microbiota makeup. Except for three studies [18,23,24], none of the other studies analyzed reported medication intake, which may have contributed to the disparities in the outcomes of this analysis.

## 6. Conclusions

According to current studies, nutrition is an important determinant in the makeup of the human gut microbiota, which is crucial for metabolizing nutrients into active postbiotics for humans. Current research suggests that switching to a plant-based diet may help increase the diversity of health-promoting bacteria in the gut. However, more research is needed to describe the connections between nutrition, the microbiome, and health outcomes because of their complexity and individual heterogeneity.

## Figures and Tables

**Table 1:** Summary characteristics of included studies (n = 12).

Study, Country, Year	Study Design	Sample Size	Participants	Mean Age (Years)	Type of Diet	Intervention Duration	Genomic Methods
Peltonen et al. [14] Finland 1994	RCT	53	Rheumatoid arthritis	VD = 53 Omnivorous diet = 56	Vegan and LV vs. omnivorous diet	13 months	Gas–liquid chromatography
Peltonen et al. [15] Finland 1997	RCT	36	Rheumatoid arthritis	VD: 49.1 Omnivorous diet = 51.6	Vegan vs. omnivorous diet	1 month	Gas–liquid chromatography
Kim et al. [17] Korea 2013	Pre–post intervention	6	Obese individuals with T2DM and/or HT	61.6	Vegan	1 month	16s-RNA sequencing and gas chromatography
David et al. [16] USA 2014	Non-RCT	10	Healthy	21–33	Plant-based vs. animal-based diet	5 days	16s-RNA sequencing and gas chromatography
Zhang et al. [18] Sweden 2018	RCT	29	Healthy	NR	OLV vs. omnivorous diet	12 weeks	16s-RNA sequencing
Pagliai et al. [19] Italy 2019	RCT	23	Overweight adults with low-to-moderate risk for CVD	58.6 ± 9.8	OLV vs. MD	12 weeks	16s-RNA sequencing and gas chromatography
Kahleova et al. [20] USA 2020	RCT	168	Overweight	Intervention = 52.9 ± 11.7. Control = 57.5 ± 10.2	Low-fat vegan diet vs. omnivorous diet	16 weeks	16s-RNA sequencing
Djekic et al. [21] Sweden 2020	RCT	31	Adults with history of IHD	67	OLV vs. isocaloric VD	4 weeks	16s-RNA sequencing and gas chromatography
Ahrens et al. [22] USA 2021	Pre–post intervention	73	Adults with HT	46.89 ± 12.38	Plant-based diet	1 week	16s-RNA sequencing
Kohnert et al. [23] Germany 2021	RCT	53	Healthy	VD = 33.2 ± 11.2 MD = 29.9 ± 9.5	Free choice vegan diet vs. free choice meat-rich diet	4 weeks	16s-RNA sequencing
Toribio-Mateas et al. [24] UK 2021	RCT	39	Healthy	37.5 ± 8.9	Plant-based meat vs. omnivorous diet	4 weeks	16s-RNA sequencing
Kahleova et al. [25] USA 2021	RCT	62	Overweight	30–76	Vegan diet vs. omnivorous diet	16 weeks	16s-RNA sequencing

RCT, randomized controlled trial; NR, not reported; T2DM, type 2 diabetes mellitus; HT, hypertension; CVD, cardiovascular disease; IHD, ischemic heart disease; OLV, ovo-lacto vegetarian diet; LV, lacto-vegetarian diet; VD, vegetarian diet; MD, Mediterranean diet.

## Data Availability

Not applicable.

## Supplementary Materials

The following supporting information can be downloaded at: https://www.mdpi.com/article/10.3390/nu15061510/s1, Table S1: PRISMA checklist; Table S2: Search strategy; Table S3: Extracted information from eligible studies; Table S4: Quality assessment for intervention studies (NIH tool); Table S5: Quality assessment for pre-post studies (NIH tool); Table S6: Quality assessment for non-randomized study (ROBINS-I tool); Table S7: The statistically increased bacterial taxa in vegans, vegetarians and omnivores; Table S8: The statistically decreased bacterial taxa in vegans, vegetarians and omnivores.
