# Supplementary material for: Effect of Plant-Based Diets on Gut Microbiota: A Systematic Review of Interventional Studies
Source: Nutrients. 2023 Mar 21;15(6):1510. doi: 10.3390/nu15061510 (PMC10057430; doi:10.3390/nu15061510)
Supplement: Supplementary file 1 [file nutrients-15-01510-s001.zip › Table S2 - Search strategy.pdf]

Table S2. Complete database search strategies

EMBASE		
**#**	**Keyword**	**Results**
1	exp Diet, Vegetarian/ or plant-based.mp.	12244
2	vegetarian.mp. or exp Vegetarians/	8168
3	vegan.mp. or exp Vegans/	2141
4	exp Gastrointestinal Microbiome/ or gut microbe.mp.	74605
5	probiotic.mp. or exp Probiotics/	47595
6	prebiotic.mp. or exp Prebiotics/	13755
7	intervention.mp.	1106828
8	1 or 2 or 3	15981
9	4 or 5 or 6	116981
10	7 and 8 and 9	75
**OVID MEDLINE**		
**#**	**Keyword**	**Results**
1	exp Diet, Vegetarian/ or plant-based.mp.	8123
2	vegetarian.mp. or exp Vegetarians/	4843
3	vegan.mp. or exp Vegans/	1074
4	exp Gastrointestinal Microbiome/ or gut microbe.mp.	26693
5	probiotic.mp. or exp Probiotics/	24033
6	prebiotic.mp. or exp Prebiotics/	7196
7	intervention.mp.	603195
8	1 or 2 or 3	9496
9	4 or 5 or 6	51671
10	7 and 8 and 9	29
**PUBMED**		
**#**	**Keyword**	**Results**
1	Search: ((plant-based[Title/Abstract]) OR (vegetairan[Title/Abstract])) or (vegan[Title/Abstract/)	10304
2	Search: ((gut micro*[Title/Abstact]) OR (probiotic*[Title/Abstact])) OR (prebiotic&[Title/Abstact])	68639
3	Search: intervention[Title/Abstact]	693567
4	Search: ((((plant-based[Title/Abstract]) OR (vegetairan[Title/Abstract])) or (vegan[Title/Abstract/)) AND (((gut micro*[Title/Abstact]) OR (probiotic*[Title/Abstact])) OR (prebiotic&[Title/Abstact])) AND (intervention[Title/Abstact])	49
**SCOPUS**		
**#**	**Keyword**	**Results**
1	(TITLE-ABS-KEY (plant-based) OR TITLE-ABS-KEY (vegetarian*) OR TITLE-ABS-KEY (vegan*))	30609
2	(TITLE-ABS-KEY (gut AND microb*) OR TITLE-ABS-KEY (probiotic*) OR TITLE-ABS-KEY (prebiotic*))	119964
3	TITLE-ABS-KEY (intervention)	1555483
4	((TITLE-ABS-KEY (plant-based) OR TITLE-ABS-KEY (vegetarian*) OR TITLE-ABS-KEY (vegan*)) AND (TITLE-ABS-KEY (gut AND microb*) OR TITLE-ABS-KEY (probiotic*) OR TITLE-ABS-KEY (prebiotic*)) AND TITLE-ABS-KEY (intervention))	102
5	((TITLE-ABS-KEY (plant-based) OR TITLE-ABS-KEY (vegetarian*) OR TITLE-ABS-KEY (vegan*)) AND (TITLE-ABS-KEY (gut AND microb*) OR TITLE-ABS-KEY (probiotic*) OR TITLE-ABS-KEY	50

	(prebiotic*)) AND TITLE-ABS-KEY (intervention)) AND (LIMIT-TO (DOCTYPE, "ar"))	
