# Supplementary material for: Effect of Plant-Based Diets on Gut Microbiota: A Systematic Review of Interventional Studies
Source: Nutrients. 2023 Mar 21;15(6):1510. doi: 10.3390/nu15061510 (PMC10057430; doi:10.3390/nu15061510)
Supplement: Supplementary file 1 [file nutrients-15-01510-s001.zip › Table S4 - Quality assesment intervention (NIH).pdf]

Table S4. Quality assessment of the intervention studies (n=9)

	Peltonen et al., 1994 [14]	Peltonen et al., 1997 [15]	Zhang et al., 2018 [18]	Pagliai et al., 2019 [19]	Kahleova et al., 2020 [20]	Djekic et al., 2020 [21]	Kohnert et al.,2021 [23]	Toribio-Mateas et al., 2021 [24]	Kahleova et al., 2021 [25]
Q1	Y	Y	N	Y	Y	Y	Y	Y	Y
Q2	NR	NR	NR	NR	Y	NR	Y	Y	Y
Q3	Y	Y	N	Y	Y	N	Y	Y	Y
Q4	Y	Y	N	N	N	NR	N	Y	N
Q5	Y	Y	N	NR	Y	NR	NR	Y	Y
Q6	N	Y	N	Y	N	Y	Y	Y	N
Q7	Y	Y	N	Y	N	Y	Y	Y	N
Q8	Y	Y	NR	Y	N	Y	Y	Y	N
Q9	Y	Y	Y	Y	N	Y	Y	Y	N
Q10	Y	Y	NR	NR	Y	Y	Y	Y	Y
Q11	Y	Y	Y	Y	Y	Y	Y	Y	Y
Q12	N	N	N	N	N	N	N	N	N
Q13	Y	Y	N	N	Y	N	N	Y	Y
Q14	Y	Y	Y	Y	Y	N	Y	Y	Y
**Quality**	**Good**	**Good**	**Poor**	**Fair**	**Fair**	**Fair**	**Good**	**Good**	**Fair**

The quality of included studies was assessed using the National Institutes of Health (NIH) Quality Assessment Tool for Intervention Studies [12]. Q, question; NR, not reported; N, no; Y, yes.

Q 1. Was the study described as randomized, a randomized trial, a randomized clinical trial, or an RCT? **Q 2.** Was the method of randomization adequate (i.e., use of randomly generated assignment)? **Q 3.** Was the treatment allocation concealed (so that assignments could not be predicted)? **Q 4.** Were study participants and providers blinded to treatment group assignment? **Q 5.** Were the people assessing the outcomes blinded to the participants' group assignments? **Q 6.** Were the groups similar at baseline on important characteristics that could affect outcomes (e.g., demographics, risk factors, co-morbid conditions)? **Q 7**. Was the overall drop-out rate from the study at endpoint 20% or lower of the number allocated to treatment? **Q 8**. Was the differential drop-out rate (between treatment groups) at endpoint 15 percentage points or lower? **Q 9.** Was there high adherence to the intervention protocols for each treatment group? **Q 10**. Were other interventions avoided or similar in the groups (e.g., similar background treatments)? **Q 11**. Were outcomes assessed using valid and reliable measures, implemented consistently across all study participants? **Q 12.** Did the authors report that the sample size was sufficiently large to be able to detect a difference in the main outcome between groups with at least 80% power? **Q 13**. Were outcomes reported or subgroups analyzed prespecified (i.e., identified before analyses were conducted)? **Q 14.** Were all randomized participants analyzed in the group to which they were originally assigned, i.e., did they use an intention-to-treat analysis?
