# Supplementary material for: Effect of Plant-Based Diets on Gut Microbiota: A Systematic Review of Interventional Studies
Source: Nutrients. 2023 Mar 21;15(6):1510. doi: 10.3390/nu15061510 (PMC10057430; doi:10.3390/nu15061510)
Supplement: Supplementary file 1 [file nutrients-15-01510-s001.zip › Table S5 - Quality assesment pre-post (NIH).pdf]

Table S5. Quality assessment for pre-post study (n=2)

	**Kim et al., 2021 [17]**	**Ahrens et al., 2021 [22]**
**Q1**. Was the research question or objective in this paper clearly stated?	Y	Y
**Q2**. Were eligibility/selection criteria for the study population prespecified and clearly described?	N	N
**Q3**. Were the participants in the study representative of those who would be eligible for the test/service/intervention in the general or clinical population of interest?	Y	Y
**Q4**. Were all eligible participants that met the prespecified entry criteria enrolled?	NR	NR
**Q5**. Was the sample size sufficiently large to provide confidence in the findings?	NR	Y
**Q6**. Was the test/service/intervention clearly described and delivered consistently across the study population?	Y	Y
**Q7**. Were the outcome measures prespecified, clearly defined, valid, reliable, and assessed consistently across all study participants?	Y	Y
**Q8**. For exposures that can vary in amount or level, did the study examine different levels of the exposure as related to the outcome (e.g., categories of exposure, or exposure measured as continuous variable)?	N	N
**Q9.** Were the exposure measures (independent variables) clearly defined, valid, reliable, and implemented consistently across all study participants?	Y	NR
**Q10.** Was the exposure(s) assessed more than once over time?	N	N
**Q11.** Were the outcome measures (dependent variables) clearly defined, valid, reliable, and implemented consistently across all study participants?	Y	Y
**Q12.** Were the outcome assessors blinded to the exposure status of participants?	NR	NR
**Q13.** Was loss to follow-up after baseline 20% or less?	NR	NR
**Q14.** Were key potential confounding variables measured and adjusted statistically for their impact on the relationship between exposure(s) and outcome(s)?	Y	Y
**Quality**	**Fair**	**Fair**

Q, question; NR, not reported; N, no; Y, yes.
