# Supplementary material for: Effect of Plant-Based Diets on Gut Microbiota: A Systematic Review of Interventional Studies
Source: Nutrients. 2023 Mar 21;15(6):1510. doi: 10.3390/nu15061510 (PMC10057430; doi:10.3390/nu15061510)
Supplement: Supplementary file 1 [file nutrients-15-01510-s001.zip › Table S6 - Quality assesment non-RCT (ROBINS-I).pdf]

Table S6. Quality assessment for non-randomized study (ROBINS-I tool)

Study: David et al [16]

The Risk of Bias In Non-randomized Studies – of Interventions (ROBINS-I) assessment tool
(version for cohort-type studies)
Version 19 September 2016

This work is licensed under a Creative Commons Attribution-NonCommercial-NoDerivatives 4.0 International License.

ROBINS-I tool (Stage I): At protocol stage

Specify the review question

Participants	American volunteers between the ages of 21–33
Experimental intervention	Consumption of plant-based diet in ad libitum for five consecutive days
Comparator	Consumption of animal-based diet in ad libitum for five consecutive days
Outcomes	Changes in α-diversity, β-diversity, and relative abundance of bacterial taxonomic groups after dietary intervention

List the confounding domains relevant to all or most studies

Age<br>Gender<br>Antibiotic consumption<br>Tobacco use<br>Alcohol consumption<br>Anthropometric measures<br>Meat quality (Grain- vs Grass-Fed)<br>Basal Metabolic Rate

List co-interventions that could be different between intervention groups and that could impact on outcomes

Coffee was allowed on the animal-based diet<br>Cheese and salami as snacks for animal-based diet

ROBINS-I tool (Stage II): For each study

Specify a target randomized trial specific to the study

Design	Crossover Study
Participants	Six male and four female American volunteers between the ages of 21–33
Experimental intervention	Consumption of a plant-based diet in ad libitum for five consecutive days
Comparator	Consumption of an animal-based diet in ad libitum for five consecutive days

Is your aim for this study…?

- ☐ to assess the effect of *assignment to* intervention
- ☐ to assess the effect of *starting and adhering to* intervention

Specify the outcome

Specify which outcome is being assessed for risk of bias (typically from among those earmarked for the Summary of Findings table). Specify whether this is a proposed benefit or harm of intervention.

Increase in an abundance of bile-tolerant microorganisms (Alistipes, Bilophila, and Bacteroides) and decrease in levels of Firmicutes (Roseburia, Eubacterium rectale, and Ruminococcus bromii). Consequently, the *Bilophila wadsworthia* increase when abiding by the animal-based diet, which may trigger inflammatory bowel disease.

Specify the numerical result being assessed

In case of multiple alternative analyses being presented, specify the numeric result (e.g. RR = 1.52 (95% CI 0.83 to 2.77) and/or a reference (e.g. to a table, figure or paragraph) that uniquely defines the result being assessed.

Figure 2

Preliminary consideration of confounders

Complete a row for each important confounding domain (i) listed in the review protocol; and (ii) relevant to the setting of this particular study, or which the study authors identified as potentially important.

**(i) Confounding domains listed in the review protocol**				
**Confounding domain**	**Measured variable(s)**	**Is there evidence that controlling for this variable was unnecessary?***	**Is the confounding domain measured validly and reliably by this variable (or these variables)?**	**OPTIONAL: Is failure to adjust for this variable (alone) expected to favour the experimental intervention or the comparator?**
Age	Age of participants	No	Yes	No information
Biological sex	Sex	No	Yes	No information
Antibiotic history	Duration of antibiotic consumption and most recent antibiotic consumption	No	Yes	Favour Comparator/ No information
Adiposity	Body Mass Index	No	Yes	No information
Bowel movement frequency	Bowel movement frequency	No	Yes	No information
Gastrointestinal health	History of GI disorders	No	Yes	Favour Comparator
Previous eating habits	Diet before recruitment to study	No	Yes	Favour experimental / Favour comparator

**(ii) Additional confounding domains relevant to the setting of this particular study, or which the study authors identified as important**				
**Confounding domain**	**Measured variable(s)**	**Is there evidence that controlling for this variable was unnecessary?***	**Is the confounding domain measured validly and reliably by this variable (or these variables)?**	**OPTIONAL: Is failure to adjust for this variable (alone) expected to favour the experimental intervention or the comparator?**
Meat quality	Grain- vs Grass-Fed Meat	No	No information	No information
Metabolic rate of individuals	Basal Metabolic Rate and Thyroid Screening	No	No information	No information
Tobacco use	No information	No	No information	No information
Alcohol consumption	No information	No	No information	No information
Supplementation regimen	Probiotic and prebiotic supplement intake prior to study recruitment	No	No information	No information

Preliminary consideration of co-interventions

Complete a row for each important co-intervention (i) listed in the review protocol; and (ii) relevant to the setting of this particular study, or which the study authors identified as important.

**(i) Co-interventions listed in the review protocol**		
Co-intervention	Is there evidence that controlling for this co-intervention was unnecessary (e.g. because it was not administered)?	Is presence of this co-intervention likely to favour outcomes in the experimental intervention or the comparator
Caffeine Intake	Coffee was allowed in the animal-based diet	No information
Sodium intake	Optional salt was allowed for added taste in the plant-based diet	No information
		Favour experimental / Favour comparator / No information

**(ii) Additional co-interventions relevant to the setting of this particular study, or which the study authors identified as important**		
Co-intervention	Is there evidence that controlling for this co-intervention was unnecessary (e.g. because it was not administered)?	Is presence of this co-intervention likely to favour outcomes in the experimental intervention or the comparator
Fermented food intake	Cheese and salami as snacks in the animal-based diet	No information
		Favour experimental / Favour comparator / No information
		Favour experimental / Favour comparator / No information

Risk of bias assessment

Responses underlined in green are potential markers for low risk of bias, and responses in red are potential markers for a risk of bias. Where questions relate only to sign posts to other questions, no formatting is used.

Signalling questions	Description	Response options
**Bias due to confounding**		
1.1 Is there potential for confounding of the effect of intervention in this study?<br>**If N/PN to 1.1:** the study can be considered to be at low risk of bias due to confounding and no further signalling questions need be considered	The authors have obtained many data sets from the participants regarding the various confounding domains faced in the study. Most of the significant confounding domains that may heavily affect the results have been considered by the authors.	N
**If Y/PY to 1.1**: determine whether there is a need to assess time-varying confounding:		
1.2. Was the analysis based on splitting participants' follow up time according to intervention received?<br>**If N/PN**, answer questions relating to baseline confounding (1.4 to 1.6)<br>**If Y/PY**, go to question 1.3.		
1.3. Were intervention discontinuations or switches likely to be related to factors that are prognostic for the outcome?<br>**If N/PN**, answer questions relating to baseline confounding (1.4 to 1.6)<br>**If Y/PY**, answer questions relating to both baseline and time-varying confounding (1.7 and 1.8)		
**Questions relating to baseline confounding only**		
1.4. Did the authors use an appropriate analysis method that controlled for all the important confounding domains?		
1.5. **If Y/PY to 1.4**: Were confounding domains that were controlled for measured validly and reliably by the variables available in this study?		
1.6. Did the authors control for any post-intervention variables that could have been affected by the intervention?		
**Questions relating to baseline and time-varying confounding**		
1.7. Did the authors use an appropriate analysis method that controlled for all the important confounding domains and for time-varying confounding?		Y

1.8. **If Y/PY to 1.7**: Were confounding domains that were controlled for measured validly and reliably by the variables available in this study?	When obtaining the antibiotic history, participants may face recall bias. Other measurements of adiposity, including waist circumference and waist-to-hip ratio were not obtained.	PY
**Risk of bias judgement**	The selection of participants based on similar age groups and sex was controlled well to avoid any confounding effect. However, adiposity can also be measured by two other methods (waist circumference and waist-to-hip ratio), which were not taken into account and factored out. Hence they may confound on effect of the intervention.	Moderate
Optional: What is the predicted direction of bias due to confounding?	Not mentioned	Favours comparator / Unpredictable

**Bias in selection of participants into the study**		
2.1. Was selection of participants into the study (or into the analysis) based on participant characteristics observed after the start of intervention?<br>**If N/PN to 2.1:** go to 2.4<br>2.2. **If Y/PY to 2.1**: Were the post-intervention variables that influenced selection likely to be associated with intervention?<br>2.3 **If Y/PY to 2.2**: Were the post-intervention variables that influenced selection likely to be influenced by the outcome or a cause of the outcome?	Volunteers that met the list of participant criteria were already selected to follow through with the intervention randomly before starting their diet arms	N
2.4. Do start of follow-up and start of intervention coincide for most participants?	Participants started and ended their diet arms at the same time: Samples were selected based on our prior 16S rRNA gene sequencing-based analysis, representing 3 baseline days and 2 timepoints on each diet	Y
2.5. **If Y/PY to 2.2 and 2.3, or N/PN to 2.4**: Were adjustment techniques used that are likely to correct for the presence of selection biases?		
**Risk of bias judgement**	Pre-selection of participants based on similar baseline characteristics and a consistent time interval between start and follow-up of intervention prevents authors from replacing actual results with biased ones	Low
Optional: What is the predicted direction of bias due to selection of participants into the study?		Favours experimental / Favours comparator / Towards null /Away from null / Unpredictable

Bias in classification of interventions		
3.1 Were intervention groups clearly defined?	The foods allowed to be consumed by participants in both the plant- and animal-based diets were clearly defined before the intervention period.	Y
3.2 Was the information used to define intervention groups recorded at the start of the intervention?	The allocation process of individual participants into plant- or animal-based diets was not mentioned. However, no randomization had to be allocated due to the crossing over of diet arms.	PN
3.3 Could classification of intervention status have been affected by knowledge of the outcome or risk of the outcome?	There was no mention or discussion of the authors' knowledge of the intervention outcome. However, the study's aim (to examine whether different specific diets may rapidly change the gut microbiota makeup in humans) shows the lack of biases of the study approach to both plant-based and animal-based diets.	N
**Risk of bias judgement**	The authors showed little to no biases to both plant-based and animal-based diets.	Low
Optional: What is the predicted direction of bias due to classification of interventions?		Favours experimental / Favours comparator / Towards null /Away from null / Unpredictable

Bias due to deviations from intended interventions		
**If your aim for this study is to assess the effect of assignment to intervention, answer questions 4.1 and 4.2**		
4.1. Were there deviations from the intended intervention beyond what would be expected in usual practice?	The participants on the vegetarian diet arm had similar results that did not deviate significantly between each other. The deviations were expected.	N
4.2. **If Y/PY to 4.1**: Were these deviations from intended intervention unbalanced between groups *and* likely to have affected the outcome?		
**If your aim for this study is to assess the effect of starting and adhering to intervention, answer questions 4.3 to 4.6**		
4.3. Were important co-interventions balanced across intervention groups?		
4.4. Was the intervention implemented successfully for most participants?		
4.5. Did study participants adhere to the assigned intervention regimen?		
4.6. **If N/PN to 4.3, 4.4 or 4.5**: Was an appropriate analysis used to estimate the effect of starting and adhering to the intervention?		
**Risk of bias judgement**	The deviations in gut microbiome analysis were expected and not beyond the anticipated ranges	Low
Optional: What is the predicted direction of bias due to deviations from the intended interventions?		Favours experimental / Favours comparator / Towards null /Away from null / Unpredictable

Bias due to missing data		
5.1 Were outcome data available for all, or nearly all, participants?	Data from all participants was obtained and included in the analysis.	Y
5.2 Were participants excluded due to missing data on intervention status?	There was no missing data on the intervention status of any participants in the study	N
5.3 Were participants excluded due to missing data on other variables needed for the analysis?	It was not mentioned that any participants were excluded due to missing data on other variables needed for the analysis	N
5.4 **If** PN/N **to 5.1, or** Y/PY **to 5.2 or 5.3**: Are the proportion of participants and reasons for missing data similar across interventions?		
5.5 **If** PN/N **to 5.1, or** Y/PY **to 5.2 or 5.3**: Is there evidence that results were robust to the presence of missing data?		
**Risk of bias judgement**	The study showed no evidence of data loss or disappearance.	Low
Optional: What is the predicted direction of bias due to missing data?		Unpredictable

Bias in measurement of outcomes		
6.1 Could the outcome measure have been influenced by knowledge of the intervention received?	Authors who carried out the stool culture sequencing could have altered the gene sequencing machine settings	N
6.2 Were outcome assessors aware of the intervention received by study participants?	Assessors were also the same people who allocated the interventions. No concealment or blinding	Y
6.3 Were the methods of outcome assessment comparable across intervention groups?	Both groups were analyzed on their stool culture in the same method	Y
6.4 Were any systematic errors in measurement of the outcome related to intervention received?	Only full-length, high-quality reads (−r=0) were used for analysis.	NI
**Risk of bias judgement**		Moderate
Optional: What is the predicted direction of bias due to measurement of outcomes?		Favours experimental / Favours comparator / Towards null /Away from null / Unpredictable

Bias in selection of the reported result		
Is the reported effect estimate likely to be selected, on the basis of the results, from...<br>7.1. ... multiple outcome *measurements* within the outcome domain?	Custom Python programs and software were used for the majority of the analysis of 16S rRNA datasets, including calculations of α- and β-diversity.	N
7.2 ... multiple *analyses* of the intervention-outcome relationship?	The presence of 16s rRna gene sequencing was followed by the functional and taxonomic analysis of the RNA sequencing data.	N

7.3 … different *subgroups*?	No information provided	NI
**Risk of bias judgement**		Low
Optional: What is the predicted direction of bias due to selection of the reported result?		Favours experimental / Favours comparator / Towards null /Away from null / Unpredictable

**Overall bias**		
**Risk of bias judgement**	Lack of missing data and measurement of outcomes as well as low bias risk when reporting results	Low
Optional: What is the overall predicted direction of bias for this outcome?		Favours experimental / Favours comparator / Towards null /Away from null / Unpredictable

This work is licensed under a Creative Commons Attribution-NonCommercial-NoDerivatives 4.0 International License.
