# Supplementary material for: Effect of Plant-Based Diets on Gut Microbiota: A Systematic Review of Interventional Studies
Source: Nutrients. 2023 Mar 21;15(6):1510. doi: 10.3390/nu15061510 (PMC10057430; doi:10.3390/nu15061510)
Supplement: Supplementary file 1 [file nutrients-15-01510-s001.zip › Table S7 - Increased abundance of taxa.pdf]

Table S7. The statistically increased bacterial taxa in vegans, vegetarians and omnivores.

Class	Family	Genus	Species	References
Clostridia	Lachnospiraceae	*Roseburia*	*Roseburia hominis*	Ahrens et al [22]; Zhang et al [18]
-	-	-	*Roseburia inulinivorans*	Zhang et al [18]
-	-	-	*Ruminococcus lactaris*	Zhang et al [18]
-	-	*Lachnospiraceae NK4A136 group*	*Lachnospiraceae NK4A136 group bacterium*	Ahrens et al [22]
-	-	*Anaerostipes*	*Anaerostipes hadrus*	Ahrens et al [22];Pagliai et al [19]
-	-	*Blautia*	*Blautia obeum*	Ahrens et al [22]
-	-	-	*Blautia faecis*	Ahrens et al [22]
Clostridia	Ruminococcaceae	*Faecalibacterium*	*Faecalibacterium prausnitzii*	Ahrens et al [22];Kahleova et al [20]; Kohnert et al [23]; Toribio et al [24]
-	-	*Subdoligranulum*	-	Ahrens et al [22]
-	-	*Ruminococcus*	-	Ahrens et al [22]; Kohnert et al [23]; Zhang et al [18]; Kahleova et al [25]
-	Monoglobaceae	*Monoglobus*	-	Ahrens et al [22]
-	-	*Veillonella*	*Veillonella dispar*	Ahrens et al [22]
-	-			Ahrens et al [22]
-	Eggerthellaceae	*Adlercreutzia*	*Adlercreutzia equolifaciens*	Ahrens et al [22]
-	Christensenellaceae	*Christensenellaceae R-7 group*	-	Ahrens et al [22]
-	Butyricicoccaceae	*Butyricicoccus*	-	Ahrens et al [22]
-	Erysipelatoclostridiaceae	-	-	Ahrens et al [22]
	Enterobacteriaceae	-	-	Ahrens et al [22]
-	-		*Bacteroides thetaiotaomicron*	Ahrens et al [22]
-	-	-	*Dorea formicigenerans*	Ahrens et al [22]
-	-	*Alistipes*	-	David et al [16]; Kohnert et al [23]
	-	*Bilophila*	-	David et al [16]

Bacteroidia	-	*Bacteroides*	*Bacteroides fragilis*	David et al [16]; Kim et at [17]; Kohnert et al [23]
	-	-	*Clostridium*	Kim et al [17]
	-	*Fusicatenibacter*	-	Djekic et al [21]
	-	*Shuttleworthia*	-	Djekic et al [21]
	-	*Akkermansia*	-	Djekic et al [21]
	-	*DTU089*	-	Djekic et al [21]
	-	*Anaerofilum*	-	Djekic et al [21]
	-	*Coprococcus*	-	Kohnert et al [23]
	-	*Dialister*	-	Kohnert et al [23]
	-	*Dorea*	-	Kohnert et al [23]
	-	*Phascolarctobacterium*	-	Kohnert et al [23]
	-	*Streptococcus*	-	Pagliai et al [19]
	-	-	*Lactobacillus plantarum*	Zhang et al [18]
	-	-	*Proteus mirabilis*	Zhang et al [18]
	Eubacteriaceae	*Eubacterium*		Kahveola et al [25]
