# Supplementary material for: Effect of Plant-Based Diets on Gut Microbiota: A Systematic Review of Interventional Studies
Source: Nutrients. 2023 Mar 21;15(6):1510. doi: 10.3390/nu15061510 (PMC10057430; doi:10.3390/nu15061510)
Supplement: Supplementary file 1 [file nutrients-15-01510-s001.zip › Table S8 - Reduced abundance of taxa.pdf]

Table S8. The statistically reduced bacterial taxa in vegans, vegetarians and omnivores.

Class	Family	Genus	Species	References
-		*Faecalibacterium*	-	Kohnert et al [23]
-		*Ruminococcus*	-	Kohnert et al [23]
-	Enterobacteriaceae	-	-	Kim et al [17]
-	Barnesiellaceae	-	-	Ahrens et al [22]
-	Sutterellaceae	-	-	Ahrens et al [22]
-	Marinifilaceae	-	-	Ahrens et al [22]
-	Desulfovibrionaceae	-	-	Ahrens et al [22]
-	Tannerellaceae	*Parabacteroides*	*Parabacteroides merdae*	Ahrens et al [22], Djekic et al [21]
-		-	*Parabacteroides distasonis*	Ahrens et al [22]
Bacteroidia	Rikenellaceae	*Alistipes*	*Alistipes putredinis*	Ahrens et al [22]
-		-	*Alistipes sp. HGB5*	Zhang et al [18]
-		-	*Alistipes shahii*	Zhang et al [18]
-		-	*Alistipes putredinis*	Zhang et al [18]
-	Acidaminococcaceae	*Phascolarctobacterium*	-	Ahrens et al [22]
Bacteroidia	Bacteroidaceae	*Bacteroides*	*Bacteroides caccae*	Ahrens et al [22]; Kohnert et al [23]; Kahleova et al [25]
-		-	*Bacteroides vulgatus*	Ahrens et al [22]
-	-	*Coprococcus*	*Coprococcus comes*	Ahrens et al [22]; Kohnert et al [23]
-	-	-	*Bilophila wadsworthia*	Ahrens et al [22]
-	-	-	*Blautia massiliensis*	Ahrens et al [22]
-	-		*Bacteroides fragilis*	Kahleova et al [20], Kahleova et al [25]
-	-	*Roseburia*	-	David et al [16]; Kohnert et al [23]
-	-	-	*Eubacterium rectale*	David et al [16]
-	-	-	*Ruminococcus bromii*	David et al [16]
-	-	*Clostridium*	-	Djekic et al [21]; Kohnert et al [23]; Pagliai et al [19]
-	-	*Ruminiclostridium 5*	-	Djekic et al [21]

-	-	*Parasutterella*	-	Djekic et al [21]
-	-	*Negativibacillus*	-	Djekic et al [21]
-	-	*Oscillospira*	-	Djekic et al [21]
-	-	*Melainabacteria*	-	Djekic et al [21]
-	-	*Akkermansia*	-	Kohnert et al [23]
-	-	*Bifidobacterium*	-	Kohnert et al [23]
-	-	*Odoribacter*	-	Pagliai et al [19]
-	-	-	*Streptococcus thermophilus*	Zhang et al [18]
